# Single-cell analysis of bovine muscle-derived cell types for cultured meat production

**DOI:** 10.3389/fnut.2023.1212196

**Published:** 2023-09-13

**Authors:** Tobias Messmer, Richard G. J. Dohmen, Lieke Schaeken, Lea Melzener, Rui Hueber, Mary Godec, Carin Didoss, Mark J. Post, Joshua E. Flack

**Affiliations:** ^1^Mosa Meat B.V., Maastricht, Netherlands; ^2^Maastricht University, Maastricht, Netherlands

**Keywords:** single-cell RNA sequencing, cultured meat, cell purification, muscle stem cells, satellite cell heterogeneity

## Abstract

Cultured meat technologies leverage the proliferation and differentiation of animal-derived stem cells *ex vivo* to produce edible tissues for human consumption in a sustainable fashion. However, skeletal muscle is a dynamic and highly complex tissue, involving the interplay of numerous mono- and multinucleated cells, including muscle fibers, satellite cells (SCs) and fibro-adipogenic progenitors (FAPs), and recreation of the tissue *in vitro* thus requires the characterization and manipulation of a broad range of cell types. Here, we use a single-cell RNA sequencing approach to characterize cellular heterogeneity within bovine muscle and muscle-derived cell cultures over time. Using this data, we identify numerous distinct cell types, and develop robust protocols for the easy purification and proliferation of several of these populations. We note overgrowth of undesirable cell types within heterogeneous proliferative cultures as a barrier to efficient cultured meat production, and use transcriptomics to identify conditions that favor the growth of SCs in the context of serum-free medium. Combining RNA velocities computed *in silico* with time-resolved flow cytometric analysis, we characterize dynamic subpopulations and transitions between active, quiescent, and committed states of SCs, and demonstrate methods for modulation of these states during long-term proliferative cultures. This work provides an important reference for advancing our knowledge of bovine skeletal muscle biology, and its application in the development of cultured meat technologies.

## Introduction

Cultured meat is an emergent technology that leverages *in vitro* proliferation and differentiation of stem cells to produce edible tissues that mimic conventional meat (1, 2). While there are numerous potential advantages to this technology, including reduced greenhouse gas emissions and improved animal welfare (3), many technical challenges remain, such as the removal of animal-derived components, scaling of culture volumes, and cost reduction (4, 5). Moreover, consumer acceptance will be reliant on the taste and texture of cultured meat products closely replicating those of the conventionally-reared equivalent (6, 7).

Meat is composed primarily of skeletal muscle, a complex tissue whose function requires the interplay of numerous mono- and multinucleated cell types, including muscle fibers, satellite cells (SCs) and fibro-adipogenic progenitors (FAPs), as well as vascular, nervous and connective tissue (8, 9). While most cultured meat products currently being pursued consist of unstructured muscle fibers, with or without fat tissue, accurate recreation of the entire tissue requires the identification, purification, proliferation and characterization of a broad range of cell types (10, 11). Descriptions of the composition of human and murine skeletal muscle at the cellular level are now available (12–16), but a similarly detailed understanding of muscle biology in agriculturally relevant species, such as cattle, is currently lacking, limiting knowledge of starting cell types for cultured meat development, as well as characterized immunophenotypes and methods for their purification. Furthermore, the extent to which complex muscle-derived cell behaviors and interactions are recapitulated during *in vitro* culture is unclear (17, 18), hindering the design of optimized bioprocesses that maximize cellular proliferation potential.

Here, we used droplet-based single-cell RNA sequencing (scRNA-seq) to profile bovine skeletal muscle, and muscle-derived cell cultures, in a time-resolved fashion across the process of cultured meat production. We use the resultant dataset to gain insight into transcriptional heterogeneity between and within cell types, and to inform various steps of cultured meat bioprocess design, including cell type selection, design of antibody panels for cell purification, and the development of selective proliferation medium formulations.

## Results

### scRNA-seq identifies 11 distinct cell types in bovine muscle

**Figure 1:**
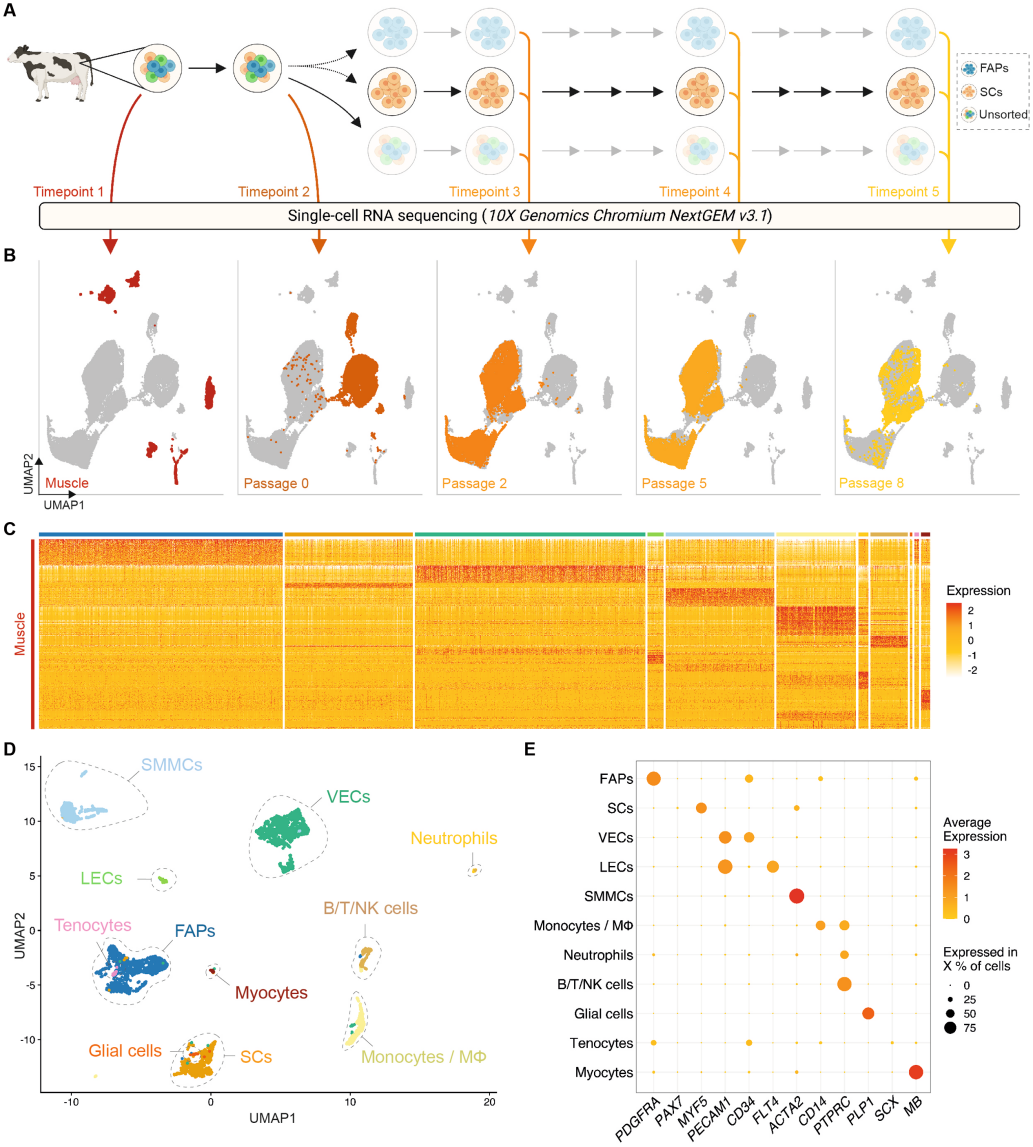
Single-cell RNA sequencing of bovine muscle and muscle-derived cell cultures. **(A)** Overview of experimental design; colored arrows and numerals indicate timepoints of RNA sequencing of single cells isolated from bovine muscle (Timepoint 1), at passage 0 after 72 h in serum-free growth medium (Timepoint 2) and at passages 2, 5, and 8 (Timepoints 3, 4, and 5 respectively); solid arrows indicate passaging, dotted arrows indicate FACS sorting; **(B)** Combined UMAP plots showing single cells (individual points) from all five experimental timepoints; clustering is based on first 30 principal components, cells from each timepoint are colored in each respective plot; **(C)** Heatmap displaying normalized expression of significantly upregulated genes (log_2_FC > 1, FDR < 0.05) in each cluster identified in Timepoint 1. **(D)** UMAP of Timepoint 1; cells are colored and clusters labeled for their assigned cell types; **(E)** Dotplot showing normalized expression of cell-type specific genes, averaged within each population; dot size indicates percentage of cells expressing the respective gene with at least 1 count.

In order to investigate heterogeneity between and within bovine muscle-derived cell types, we used the 10x Genomics Chromium platform for scRNA-seq to study gene expression at five timepoints in a primary adult stem cell-based cultured meat production process (Figure 1A; Table 1). A total of 36,129 single-cell transcriptomes, with an average expression of 3,815 genes and 21,131 transcripts per cell, were analyzed across 10 donor cattle and assembled into a single dataset (Supplementary Figure S1). Using uniform manifold approximation and projection (UMAP) dimensionality reduction, based on the first 30 principal components (Figure 1B), cells from Timepoints 1 (muscle tissue) and 2 (passage 0, after 72 h *in vitro* culture) clustered separately from each other, and from Timepoints 3 to 5 (after passages 2, 5, and 8 respectively), indicating significant transcriptomic changes between timepoints and within cell types during the proliferative process.

**Table 1:** scRNA-seq metadata.

Animal /Genotype	Breed	Sex	Age	Sorted population	Number of cells				
					Timepoint 1	Timepoint 2	Timepoint 3	Timepoint 4	Timepoint 5
					Muscle	P0	P2	P5	P8
01	Belgian Blue	Female	Adult	Unsorted	249	905	927	801	443
02	Belgian Blue	Female	Calf	Unsorted	446	783	1,102	998	216
03	Belgian Blue	Female	Adult	N/A	230	–	–	–	–
04	Belgian Blue	Female	Calf	FAPs	735	1,058	917	1,355	317
05	Belgian Blue	Female	Calf	SCs	754	1,001	1,144	1,041	239
06	Belgian Blue	Male	Adult	SCs	362	1,270	1,092	1,522	567
07	Belgian Blue	Female	Calf	FAPs	458	1,136	915	1,177	307
08	Belgian Blue	Male	Adult	FAPs	363	1,118	863	1,442	316
09	Belgian Blue	Female	Adult	Unsorted	232	872	905	922	159
10	Belgian Blue	Male	Calf	SCs	566	1,448	1,101	1,147	208
				Total	4,395	9,591	8,966	10,405	2,772
								Cumulative	36,129

Within enzymatically digested bovine muscle tissue (Timepoint 1), we identified 11 defined populations of mononuclear cells with distinct gene expression profiles (Figure 1C; Supplementary Table S1), which were present in varying proportions in all donor animals (Supplementary Figure S2A). Based on differential gene expression, these clusters were assigned to cell types previously characterized in other species (12, 13, 19), namely fibro-adipogenic progenitors (FAPs), satellite cells (SCs), vascular and lymphatic endothelial cells (ECs; vascular, VECs; lymphatic, LECs, Supplementary Figures S2B,C), smooth muscle and mesenchymal cells (SMMCs), monocytes/macrophages (MΦ), neutrophils, lymphocytes (B/T/NK cells), glial cells, tenocytes and myocytes (Figure 1D; Supplementary Figure S2D). The expression of canonical genes in each of these populations showed notable similarity to that observed in analogous murine studies (Figure 1E; Supplementary Figure S2E) (14).

### Cell types can be identified *in vitro* by unique surface marker expression

**Figure 2:**
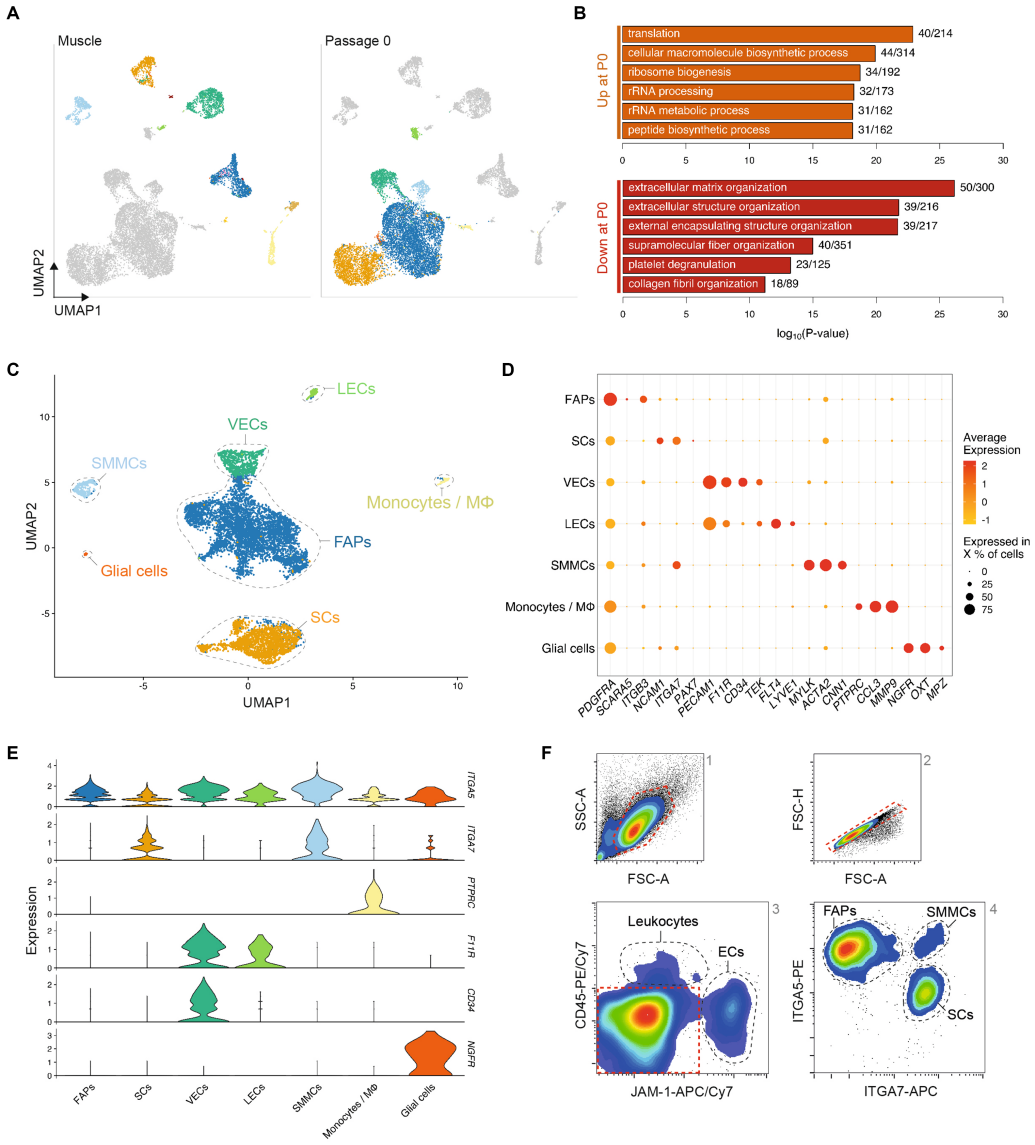
Transcriptomic analysis defines adherent cell types with distinct immunosurface phenotypes. **(A)** Combined UMAP of Timepoints 1 and 2, colored for populations in Timepoint 1 (left) or 2 (right), respectively; **(B)** Top five most significantly enriched GO terms corresponding to differentially expressed genes up- (top) or downregulated (bottom) between Timepoints 1 and 2, after regressing out the effect of cell type; **(C)** UMAP of Timepoint 2; cells are colored and clusters labeled for their assigned cell types; **(D)** Normalized gene expression of canonical markers in each population at Timepoint 2; **(E)** Violin plots showing expression of surface markers in different cell types at Timepoint 2; **(F)** Representative flow cytometry contour plots showing gating strategy for purification of cell types; red dashed lines indicate gates for the subsequent plot (from 1 to 4); black dashed lines indicate sorting gates for the labeled populations.

To identify proliferative muscle tissue-derived cell types that might be used for cultured meat production, we analyzed the adherent cell fraction after 72 h (Timepoint 2) culture in serum-free growth medium (SFGM, Supplementary Table S2). We discerned 7 populations present in these cultures, which were separated from the same populations in Timepoint 1 by UMAP, indicating stark transcriptional changes during the transition to *in vitro* culture (Figure 2A). Gene Ontology (GO) terms corresponding to differentially expressed genes (independent of cell type) between Timepoints 1 and 2 (Figure 2B) suggest that these transcriptional switches relate to decreased interaction with the extracellular matrix (ECM) and increased protein production, concomitant with increased cellular proliferation in the *in vitro* environment.

Comparison of Timepoints 1 and 2 enabled the assigning of cell identities to individual clusters (Figure 2C), indicating that of the 11 populations identified in Timepoint 1, only myocytes, tenocytes, neutrophils and lymphocytes did not persist in short-term serum-free culture. Conversely, FAPs, SCs, SMMCs, ECs, glial cells and monocytes/macrophages remained present in cultures from all genotypes (donor animals) sequenced (Supplementary Figures S3A,B). These were the same cell types we identified by scRNA-seq when culturing cells in growth medium (GM) containing 20% fetal bovine serum (FBS; Supplementary Figure S3C; Supplementary Table S2), indicating that our SFGM formulation was able to support culture of muscle-derived cells as robustly as traditional serum-containing media. Cell populations showed similar gene expression profiles between serum and serum-free conditions (Supplementary Figure S3D), and were present in comparative ratios (Supplementary Figure S3E).

Analysis of differentially expressed genes yielded highly and exclusively expressed markers for each population (Figure 2D) (10, 20) that have previously been described in mouse and human (12–14, 21). Filtering these lists for genes encoding plasma membrane-localized proteins facilitated the identification of candidate cell surface markers for separation of populations by flow cytometry (Figure 2E). Staining of muscle tissue-derived cells 72 h post-isolation for JAM-1 (*F11R*), CD45 (*PTPRC*), ITGA7 and ITGA5 confirmed the suitability of this quartet of markers as a FACS panel (Figure 2F; Supplementary Figure S3F).

### Four principal muscle-derived cell types can proliferate *in vitro*

**Figure 3:**
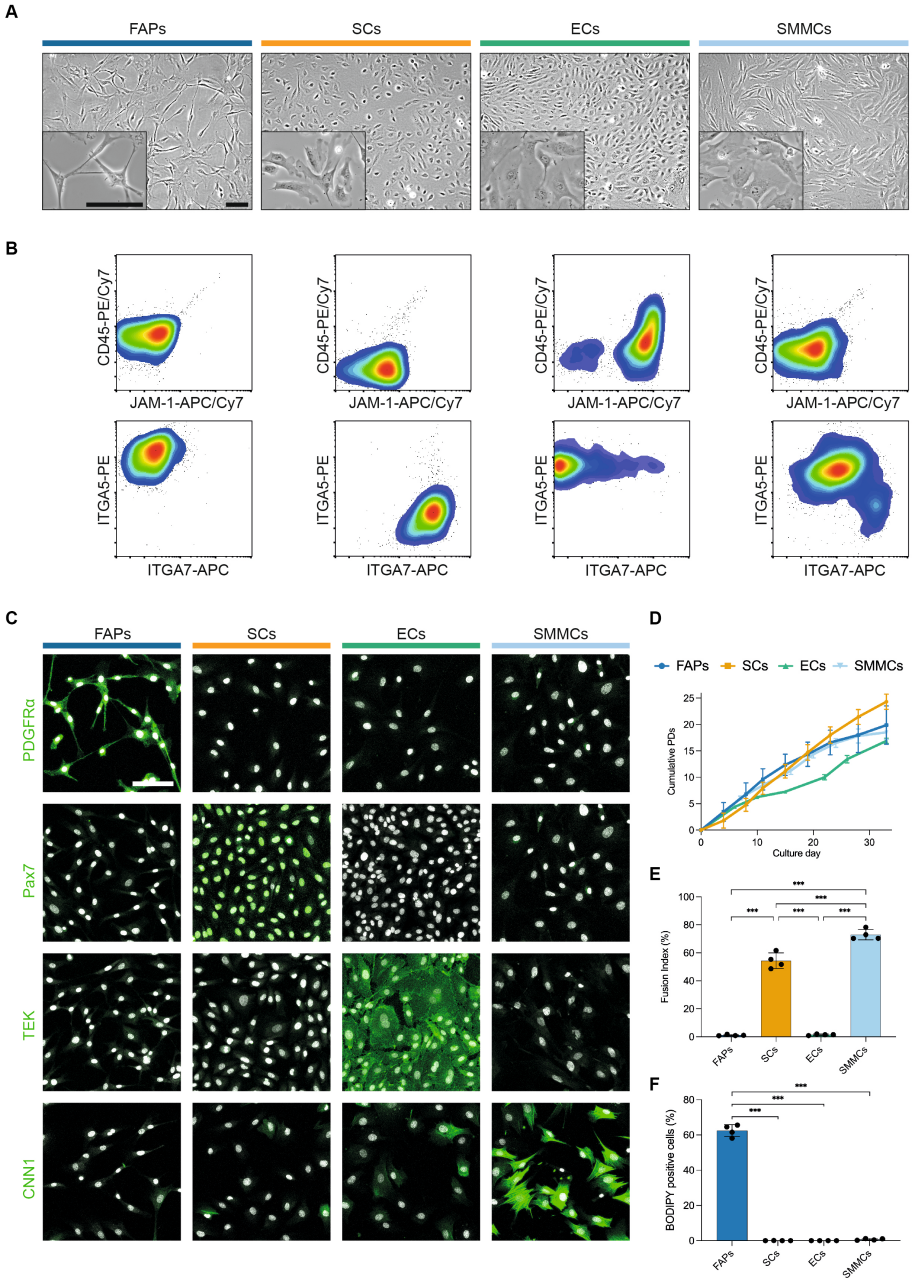
Four muscle-derived cell types can be purified and expanded in long-term culture. **(A)** Brightfield microscopy images of purified FAPs (blue), SCs (orange), ECs (green) and SMMCs (light blue) *in vitro*; scale bars = 100 μm; **(B)** Flow cytometry plots of purified cell types stained for CD45/JAM-1 (top) and ITGA5/ITGA7 (bottom) immediately after FACS; **(C)** Immunofluorescent staining for PDGFRα, Pax7, TEK, and CNN1 (green) in purified cell types; nuclei stained with Hoechst (white); scale bar = 100 μm; **(D)** Long-term growth curves of purified cell types *in vitro*, shown as mean cumulative population doublings (PDs), *n* = 3; **(E)** Fusion indices of purified cell types after 72 h of myogenic differentiation in SFDM, *n* = 4; **(F)** Percentage of BODIPY-positive cells in purified cell types after 240 h of adipogenic differentiation in serum-free adipogenic differentiation medium, *n* = 4; Error bars indicate standard deviation (*SD*). Adjusted *p*-values: * < 0.05, ** < 0.01, *** < 0.001.

In order to characterize their *in vitro* phenotypes in more detail, we next sorted muscle-derived cells into FAPs, SCs, ECs and SMMCs using the FACS panel previously described (Figure 2). The purified populations exhibited markedly different morphologies; while FAPs had a spindle-like morphology and SCs were more spherical, ECs and SMMCs appeared flatter and larger (Figure 3A). Flow cytometric analysis immediately post-FACS using the same antibody panel confirmed high sorting purities for all populations (Figure 3B). In addition, immunofluorescent staining for canonical markers PDGFRα, Pax7, TEK (also known as Tie2), and Calponin-1 (CNN1) further verified the identity of FAPs, SCs, ECs, and SMMCs, respectively (Figure 3C).

Long-term proliferation of each of these populations was supported in SFGM (FAPs, SCs) or BioAMF-3 medium (ECs, SMMCs), with each culture undergoing at least 15 population doublings (PDs) over a period of 6 passages (Figure 3D). Proliferation rates varied between cell types (for example, from 0.46 (ECs) to 1.0 PDs/day (SMMCs) at early passage), and tended to decrease over time (Supplementary Figure S4A).

We next sought to functionally characterize the four purified cell populations by performing myogenic and adipogenic differentiation assays. SCs demonstrated robust myogenic differentiation, as measured by fusion index (53.3 ± 5.6%), but interestingly SMMCs also showed pronounced fusion (73.0 ± 3.7%, Figure 3E; Supplementary Figure S4B), as previously described in mice (12). Expectedly, adipogenic differentiation occurred exclusively in FAPs (Figure 3F; Supplementary Figure S4C). Both media formulations supported short-term cell survival, although cell counts were significantly lower for SCs and SMMCs in a serum-free adipogenic differentiation medium (*p* < 0.001; Supplementary Figures S4D,E).

### Optimized culture conditions prevent SC overgrowth by FAPs

**Figure 4:**
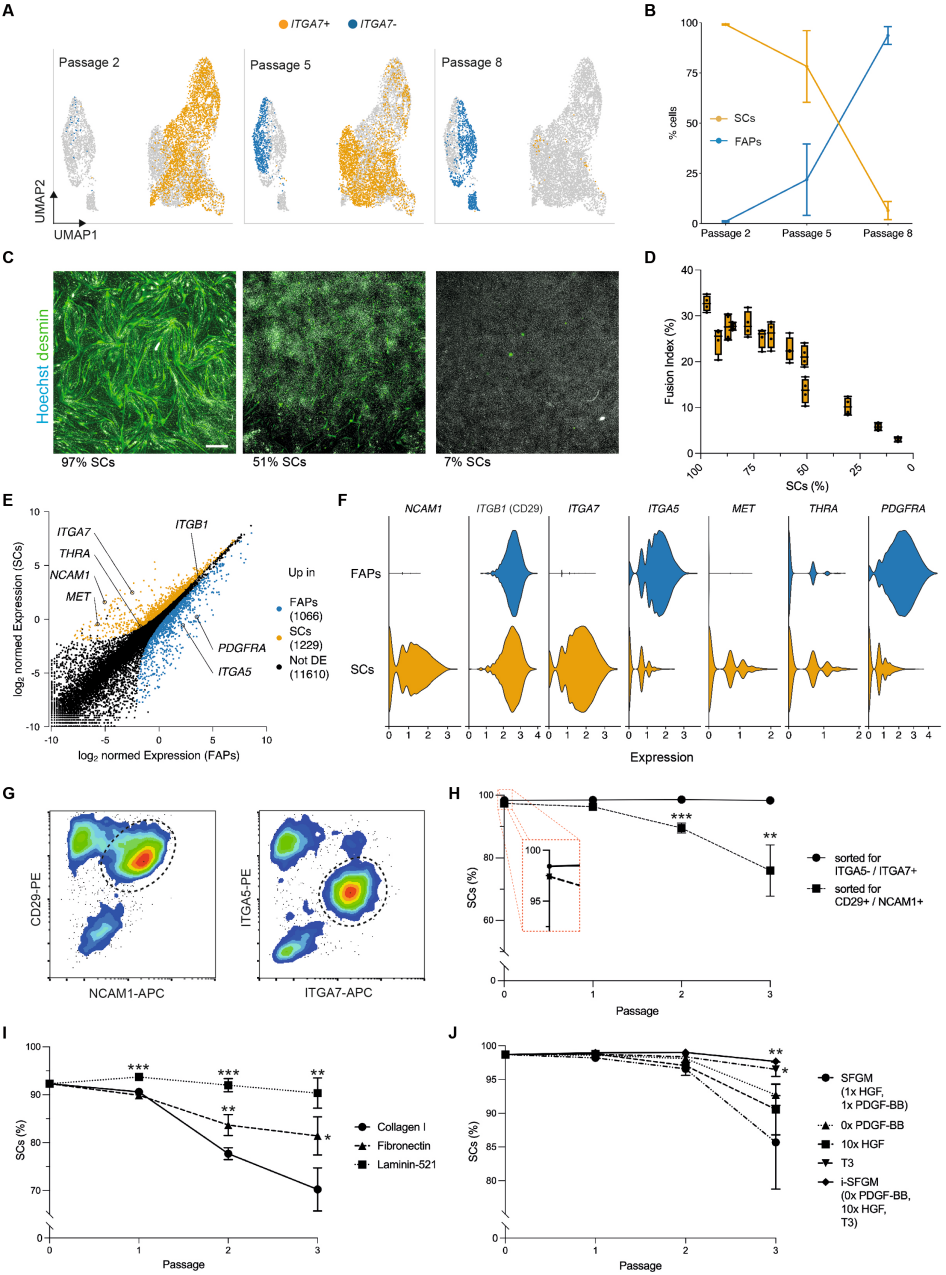
Optimized culture conditions prevent overgrowth of SCs. **(A)** Combined UMAPs of sorted SCs at passages 2 (Timepoint 3, left), 5 (Timepoint 4, center) and 8 (Timepoint 5, right); clusters in respective passages are colored for *ITGA7* expression; **(B)** Proportion of SCs (*ITGA7*+) during long-term culture; error bars indicate *SD*, *n* = 3; **(C)** Immunofluorescent staining of cultures with varying proportions of SCs and FAPs for desmin (green) and Hoechst (white) after 72 h of myogenic differentiation in SFDM; scale bar = 100 μm; **(D)** Fusion indices in varying proportions of SCs and FAPs after 72 h of myogenic differentiation in SFDM corresponding to **(C)**, shown as box plots, *n* = 4; **(E)** Normalized gene expression in SCs and FAPs at passage 2 (Timepoint 3). Points represent genes, which are colored if significantly (log_2_-FC > 1, FDR < 0.05) upregulated in FAPs (blue) or SCs (orange). Selected genes are annotated; **(F)** Violin plots showing expression of differentially expressed receptors in SCs and FAPs at passage 2; **(G)** Flow cytometry plots of muscle-derived cells after 72 h in SFGM stained for CD29/NCAM1 (left) or ITGA5/ITGA7 (right) prior to FACS; cells are shown after removal of doublets and CD45+/JAM-1+ cells; dashed lines indicated gating strategy for sorting; **(H)** Proportion of SCs purified with FACS protocols shown in **(G)** during long-term culture, as measured by flow cytometry; *t*-test was performed; *n* = 3; **(I)** Proportion of SCs during long-term culture on different coatings as indicated. *t*-tests were performed against Collagen control, *n* = 3; **(J)** As **(I)**, but for different media formulations as indicated, where SFGM serves as control, *n* = 3; Error bars indicate SD. Adjusted *p*-values: * < 0.05, ** < 0.01, *** < 0.001.

We next aimed to understand heterogeneity within FACS-purified SC cultures during long-term proliferation. Surprisingly, we found that cells in these cultures clustered into two distinct populations, marked by differential expression of *ITGA7* (Figure 4A). *ITGA7*- cells expressed FAP marker genes such as *ITGA5* (Supplementary Figure S5A; Figure 2), suggesting that these cells were FAPs that were inaccurately sorted into, and remained prevalent within, SC cultures. Quantification of these cell types showed that the proportion of FAPs increased over time (from 1.0 to 93.6% within 6 passages), indicating that our standard culture conditions favored long-term proliferation of FAPs over SCs (Figure 4B; Supplementary Figures S5B,C). The percentage of contaminating FAPs correlated negatively with myogenic differentiation, as determined by fusion index (*R*^2^ = 0.91, *p* < 0.001, Figures 4C,D), emphasizing the importance of eliminating overgrowth by non-SCs for cultured muscle production.

We therefore sought to adapt the long-term culture conditions, with the aim of preventing FAPs from overgrowing SCs. To inform our approach, we filtered the scRNA-seq data for differentially expressed genes between SCs and FAPs encoding receptors that interact with proteins commonly used as surface coatings or growth factors, revealing a number of interesting candidates (Figures 4E,F). Sorting SCs using an ITGA7+/ITGA5- strategy decreased FAP contamination as compared to a CD29+/NCAM1+ sorting strategy [used in previous studies (22)], significantly reducing FAP overgrowth after three passages (*p* = 0.002; Figures 4G,H). Culturing SCs contaminated with FAPs on different coatings, we observed that within 3 passages, SC purity was reduced from 92.3% to 70.2 ± 4.5% on collagen I and to 81.4 ± 4.0% fibronectin (interacting with ITGA5), but remained at 90.3 ± 3.1% on laminin-521 (interacting with ITGA7; Figure 4I). We further adapted SFGM by adding triiodothyronine (T3, 30 nM, ligand for THRA), increasing the concentration of HGF (ligand for c-Met) and removing PDGF-BB (ligand for PDGFRα; Figure 4J). Each individual medium adaptation reduced FAP overgrowth over 3 passages, while the combination of all three significantly improved SC proportions compared to SFGM control (*p* = 0.008). The combined media formulation was thus labeled “improved SFGM” (i-SFGM). Interesting, FAP overgrowth was accelerated when cells were cultured at higher confluence (Supplementary Figure S5D), indicating potential differences in contact inhibition phenotypes between cell types.

### SCs transition between three dynamic states

**Figure 5:**
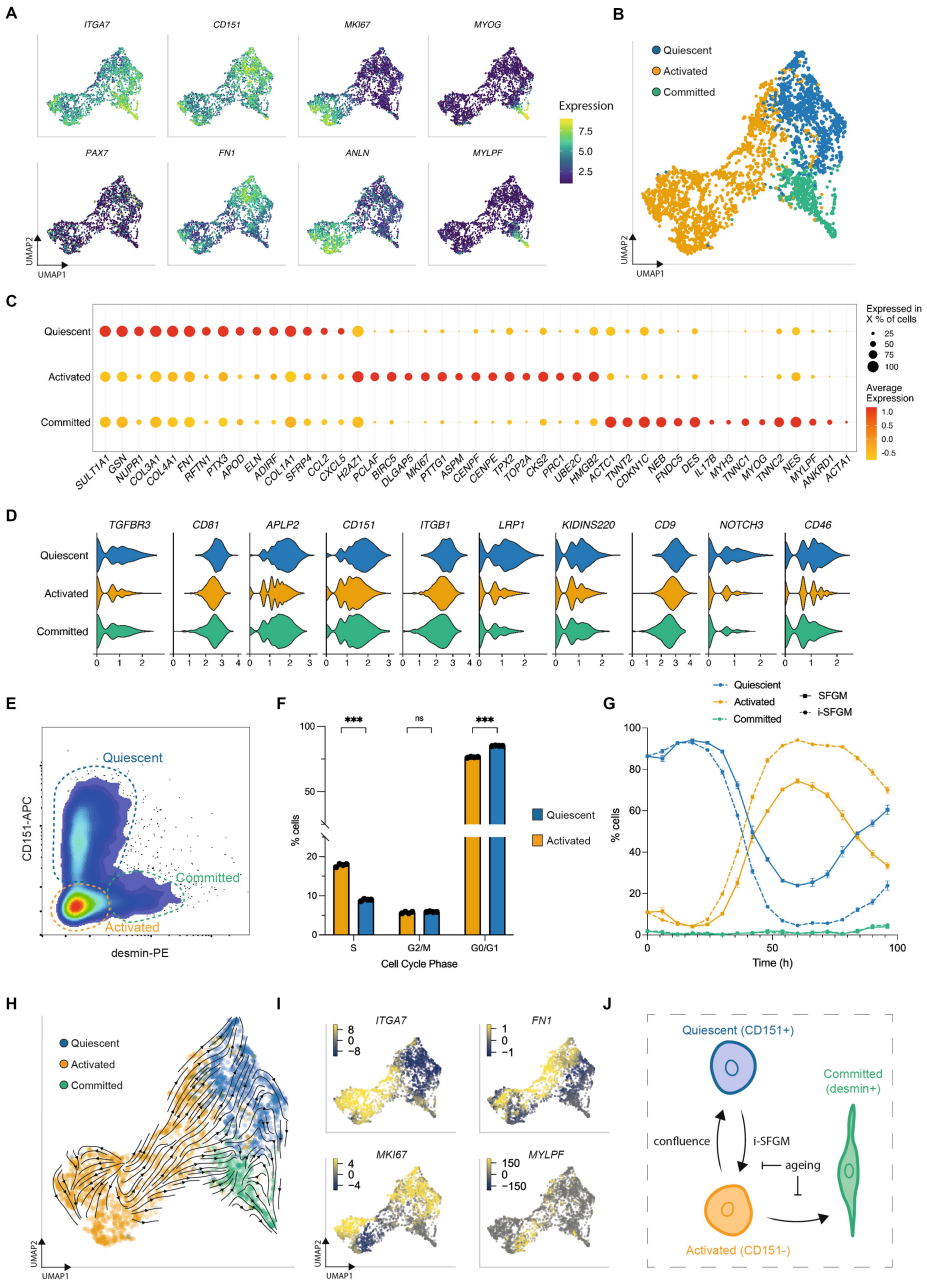
SCs switch dynamically between three cellular states *in vitro*. **(A)** UMAPs of SCs at passage 2 (Timepoint 3), colored by expression of indicated genes; **(B)** UMAP as **(A)**, but labeled according to SC state; **(C)** Normalized expression of the 15 most upregulated genes, averaged within SCs in each state; **(D)** Average expression of genes encoding quiescence-associated surface receptors in each SC state; **(E)** Flow cytometry plot of purified SCs stained for CD151 and desmin; dashed gates indicate the respective cell states; **(F)** Cell cycle analysis of CD151+ (blue) and CD151- cells (orange) determined via flow cytometry using the gating strategy in Supplementary Figure S5E; **(G)** Proportion of activated, quiescent and committed SCs over one passage in SFGM or i-SFGM media, as determined via flow cytometry; **(H)** UMAP as **(B)**, with averaged RNA velocity vectors embedded; **(I)** UMAPs as **(A)**, colored by RNA velocities of denoted genes; **(J)** Dynamic model of SC states *in vitro*; arrows indicate direction (s) of proposed transitions between states; Adjusted *p*-values: * < 0.05, ** < 0.01, *** < 0.001.

To further investigate SC heterogeneity *in vitro*, we filtered out FAPs and reanalyzed the remaining single-cell transcriptomes at passage 2 (Timepoint 3), where significant numbers of SCs remained. Reclustering identified three subpopulations within SCs, with distinct gene expression profiles suggestive of quiescent, activated, and committed states (similar to those previously observed in human and murine SCs (13, 14); Figures 5A,B). Genes encoding proteins related to focal adhesion and ECM organization were upregulated in the quiescent state, cell cycle and replication in the activated state, and myogenic differentiation the committed state (Figure 5C; Supplementary Figure S6A). Surface receptor expression in the quiescent state was similar to that previously observed by scRNA-seq in a quiescent subpopulation (therein referred to as “MuSC1”) of human SCs (Figure 5D) (13). These cell states were also observed at passages 5 and 8, but in different ratios (Supplementary Figures S6B,C), suggesting the possibility of transitioning between states.

We next sought to establish a protocol to measure the quiescent state *in vitro*. Analysing surface receptor expression in our dataset, together with the aforementioned human scRNA-seq dataset, identified CD151 as a potential marker for the quiescent population (Figure 5D). Staining for CD151 and for desmin (upregulated in committed SCs) allowed the simultaneous quantification of all three states via flow cytometry (Figure 5E; Supplementary Figure S6D). Combining this panel with EdU-staining, we confirmed increased cell cycle activity in activated as compared to quiescent SCs (Figure 5F; Supplementary Figure S6E), although the quiescent population did still show evidence of cell cycle activity. Investigating the dynamics of these subpopulations during a single passage, we found dynamic conversion between quiescent (CD151+) and activated (CD151-) states, with an initial increase in quiescent SCs followed by a rapid decline and a renewed increase as cells approach confluence (Figure 5G). The proportion of cells in the committed state remained stable throughout the first 72 h, increasing after 96 h. Investigating over multiple passages revealed that this interchangeability decreased over time (Supplementary Figure S6F). We observed that i-SFGM increased the proportion of activated SCs compared to SFGM, both within a single passage (Figure 5G) and over the course of multiple passages (Supplementary Figure S6F). In addition to transitions between cell states changing over the course of cell aging, transcriptional changes related to increased ECM remodeling and decreased translation were also observed *within* states over time (Supplementary Figure S7).

Finally, we performed RNA velocity analysis of SCs at passage 2, in order to investigate these cell state transitions *in silico*. RNA velocity averaged across all genes indicated transitions from the activated to the quiescent state or committed states (Figure 5H), while an increase in expression of cell cycle genes within a subset of quiescent cells suggested they are able to return to the activated state (Figure 5I). Taken together with *in vitro* data, this suggests a dynamic, reversible transition between activated and quiescent states, while activated cells can also differentiate toward committed myocytes as SCs become confluent (Figure 5J).

## Discussion

The complexity of muscle tissue arises from the interplay of multiple cell types (8, 9). Here, we have presented an annotated scRNA-seq dataset comprising over 36,000 muscle cellular transcriptomes, from 10 donor cattle, across five timepoints during a cultured beef production process. This dataset gives significant insight into the cellular heterogeneity present in bovine skeletal muscle for the first time, including comprehensive analysis of 11 defined cell types (Figure 1). Our analysis showed notable similarity between bovine muscle and analogous approaches employed for more well-studied species, with expression of canonical genes for distinct cell types largely conserved between cattle, human and mouse. Comparison of bovine muscle with the corresponding cell cultures shed light on transcriptional changes that occur during the transition to *in vitro* culture. Notably, genes related to protein synthesis were strongly upregulated (suggesting cell activation), while those relating to ECM were downregulated, highlighting the pivotal role of cell-matrix interactions in the muscle niche. However, despite the absence of signaling from the microenvironment, the expression of most canonical marker genes was conserved *in vitro* (Figure 2), suggesting that muscle-derived cell cultures are able to recapitulate *in vivo* behavior to a large extent.

Differential expression analysis allowed us to identify surface marker panels for physical separation of multiple cell types, including SCs, FAPs, ECs, and SMMCs (together representing over 80% of mononucleated cells). Notably, inclusion of ITGA5 in the panel offered significant purity improvements over previous protocols, as this integrin serves as a negative selection marker for SCs, while ITGB1/CD29 (previously used as a SC marker (22, 23)) is highly expressed by both SCs and FAPs. While we were able to proliferate each of these cell types for multiple passages (Figure 3), further work is needed to assess the potential of ECs and SMMCs, with respect to their long-term proliferation in the absence of FBS and their capacity to promote myogenesis or adipogenesis, for example in co-culture systems (12, 24). Other low-abundance cell types, such as macrophages, could also offer benefits in terms of tissue remodeling and angiogenesis for structured cultured meat products (25, 26).

Cultured meat production requires an extensive proliferation phase, with many PDs, to achieve required cell yields (5). This prompts several issues, including reduced growth rates, loss of differentiation capacity, and the possibility of overgrowth of undesired cell types. Transcriptomic analysis indeed revealed that sorted SCs were rapidly overgrown by a small proportion of contaminating FAPs, indicating variable growth dynamics in our initial culture conditions that favor FAP proliferation (Figure 4). While high levels of animal-derived sera have traditionally been used to control fibroblastic overgrowth in SC cultures (27), this phenomenon is poorly understood, and in any case is unsuited for cultured meat applications. We used differential expression analysis to inform several improvements to the SC culture conditions, including the switch to an ECM mimic that corresponds to the integrin expression profile of SCs [laminin is known to interact with ITGA7/ITGB1 dimers (28)], and the addition of growth factors, such as HGF, whose receptor expression is specific to SCs (29, 30). These adjustments led to a significant decrease in the rate of FAP overgrowth, although it is likely that our scRNA-seq data could inform further improvements still (such as the inhibition of cell-type specific pathways using small molecule inhibitors). Our dataset will also provide insights into design of selective media for other cell types, such as ECs, which is likely complicated by their slow proliferation. Detailed metabolic profiling, which was beyond the scope of this study, could also help to inform the design of feeding strategies that afford efficient and selective growth of desired cell types (31). Understanding and solving these overgrowth-type issues could allow for longer proliferation phases at higher production volumes. It should be noted that the experiments described here were conducted at lab scale using static 2D cultures, and that proliferation in high-density, dynamic bioreactor systems used for upscaled cultured meat production will introduce additional physical and metabolic stresses. These might act differentially across cell types or states, and could thus be additional levers to reduce overgrowth, but they might also promote formation of unfavorable cell states that we did not observe in 2D (32). Further cost reduction will also be required for industrial scale cultured meat production, and our dataset can assist in the replacement of full-length growth and ECM attachment factors with animal-free peptide alternatives with retained (or superior) cell type selectivities, proliferation rates and differentiation performance (33).

Our transcriptomic analysis also revealed remarkable heterogeneity *within* cell types, notably between distinct subpopulations of SCs, that points toward a dynamic equilibrium of quiescent, active and committed states (Figure 5). Different SC states have previously been proposed, both from dissociated muscle samples (15, 34–36) and *in vitro* cultures (16, 37, 38), although without clear consensus. We identified CD151 [previously also observed in quiescent SCs in human skeletal muscle (13)] as a cell-surface marker for the quiescent cluster, although the extent to which the quiescent phenotype we observed *in vitro* reflects physiological SC behavior during embryonic development or wound healing certainly remains unclear. Cell cycle analysis demonstrated that the ‘quiescent’ cells still showed a low level of cell cycle activity, and this label may not be fully appropriate. We also did not distinguish such states in our bovine muscle samples, perhaps because the majority of cells are activated during tissue dissociation (34). RNA velocity analysis suggests a model in which only activated SCs are able to differentiate, which is congruent with a physiological model of wound healing in which activated SCs either differentiate to form muscle fibers, or return to a quiescent state in order to preserve the ability of the tissue to respond to future regenerative stimuli. However, higher resolution velocity analysis of SCs would be informative, given that we only sampled a snapshot within each passage (when cells were approaching confluency). Indeed, the role that confluency plays in the promotion of differentiation, and the extent to which premature differentiation might negatively affect a proliferative culture, requires further study in the context of a cultured meat bioprocess design. Similarly, while increased culture length clearly affects the transitions of SCs between states, it is important to note that we also observed substantial transcriptomic differences between equivalent states at different timepoints (Supplementary Figure S7), indicating that cellular aging cannot be understood solely in terms of subpopulation ratio changes. Indeed, our data supports the hypothesis that a reduced rate of switching between states could be a characteristic of SC aging or senescence (39, 40). Future transcriptomic experiments, including single-nuclei approaches, could illuminate the formation of cultured muscle during the differentiation of SCs in 3D tissue constructs. Furthermore, although not specified in this study, it is likely that similar cellular heterogeneity is present in other cell types, including FAPs, where adipogenic and fibroblastic fate decisions could be critical for cultured fat production (41, 42). Although skeletal muscle is a well conserved tissue, some degree of species-specific heterogeneity between and within cell types should certainly be expected, and scRNA-seq approaches for other relevant organisms (including avian and fish species) will be insightful, although these may be complicated by the absence of reference genomes in some cases.

In conclusion, scRNA-seq is a powerful approach to study cell heterogeneity in the context of cultured meat production. Our dataset offers a number of important insights for cell purification and proliferation steps, has led to the development of refined medium conditions, and represents a useful resource for further analysis and improvement of cultured meat bioprocesses.

## Materials and Methods

### Cell isolation

Cells were isolated from semitendinosus muscles of 10 Belgian Blue cattle (Table 1) by collagenase digestion (CLSAFA, Worthington; 1 h; 37°C), filtration with 100 μm cell strainers, red blood lysis using a Ammonium-Chloride-Potassium (ACK) lysis buffer, and final filtration with 40 μm cell strainers. Cells from each donor animal were plated as separate cultures in serum-free growth medium (43) (SFGM, Supplementary Table S2) on fibronectin-coated (4 μg cm^−2^ bovine fibronectin, F1141, Sigma-Aldrich) tissue culture vessels for 72 h prior to FACS, or (where denoted) in growth medium (GM) containing 20% fetal bovine serum (heat-inactivated FBS; 10500-064, Gibco).

### Cell culture

Tissue culture vessels (Greiner) were coated with bovine fibronectin (4 μg cm^−2^, F1141, Sigma-Aldrich) for unsorted cells, ECs, and SMMCs, recombinant laminin-521 (0.5 μg cm^−2^, LN521-05, Biolamina) for SCs, or bovine skin collagen I (0.6 μg cm^−2^, C2124, Sigma-Aldrich) for FAPs for 1 h prior to plating, without any additional pre-treatment. Unsorted cells, SCs and FAPs were cultured in SFGM (except where otherwise noted), and passaged every 3–4 days upon reaching confluency. ECs and SMMCs were cultured in BioAMF-3 (01-196-1A, Sartorius).

To assess long-term proliferation, cells were cultured as described above for each cell type. Cells were harvested upon approaching confluence, counted, reseeded at 5 × 10^3^ cm^−2^ and purity assessed at each passage via flow cytometry.

### Single cell RNA-sequencing

#### Cell harvesting

Five timepoints throughout long-term *in vitro* culture were selected for single-cell RNA sequencing (scRNA-seq), for each of which cells from all genotypes (donor animals) were pooled in equal ratios and washed with 1% BSA in PBS prior to injection. Timepoints 1 to 5 corresponded to unsorted cells directly after isolation (“Muscle”) and after 72 h (“passage 0”), and to cultured cells (unsorted cells, SCs and FAPs) at passages 2, 5, and 8, respectively (Figure 1A; Table 1).

#### Library preparation and sequencing

25,000 cells were injected for each timepoint into a Chromium Single Cell Controller, emulsified with bar-coded gel beads and libraries were constructed following the protocol of the Chromium NextGEM Single Cell 3′ Kit V3.1 (10x Genomics). Quality control of the DNA library was performed on Qiaxcel (QIAgen) and quantified by qPCR using the KAPA SYBR Fast qPCR Master Mix (Illumina). Paired-end single cell 3′ gene expression libraries were sequenced on a Novaseq 6,000 System (Illumina) using a NovaSeq S1 flow-cell to a depth of at least 3.5 × 10^8^ reads/timepoint.

#### Data processing and demultiplexing

Raw base call files were demultiplexed using the cellranger mkfastq function (CellRanger 6.0.1). Reads were aligned to *Bos Taurus* genome (build ARS-UCD1.2) with Ensembl annotations (release 101), using CellRanger count function. Default filtering parameters of CellRanger were applied to obtain a gene expression matrix. Genotypes were deconvolved and assigned to individual cells using demuxlet (44), based on VCF files previously generated by genotyping of each donor animal with a BovineSNP50 v3 DNA Analysis BeadChip (Illumina).

#### Quality control and normalization

Across the five timepoints, 36,129 cells (90.8%) passed quality control (within 3 median absolute deviations of the median for expressed genes, total counts and percentage mitochondrial genes; Supplementary Figure S1). Counts for each timepoint were normalized using the sctransform function of Seurat with default parameters (45, 46), regressing out percentage of mitochondrial genes, library size, number of genes, and cell cycle effects.

#### Dimensionality reduction and differential gene expression analysis

Cells were clustered using the FindNeighbors() and FindClusters() functions based on the first 50 principal components and with a resolution of 0.1, respectively. Dimensionality was reduced by uniform manifold approximation and projection (UMAP) (47) using the first 30 principal components as input, 50 neighboring points and a minimal distance of 0.1.

Differentially expressed (DE) genes were computed for clusters identified in each timepoint by using the FindAllMarkers(), or between denoted conditions using FindMarkers(), with a log_2_ fold-change (log_2_-FC) threshold of 1, a false discovery rate (FDR) of 0.05, and expressed in at least 50% of cells. For Timepoint 1, cell types were assigned to each cluster based on DE genes and on predicted phenotypes from FindTransferAnchors() (48) using analogous murine scRNA-seq data [GSE143437 (14)]. ECs were further characterized by their expression of signatures derived from the Descartes human genes expression atlas (19) using the AddModuleScore function. For Timepoint 2, surface markers among the DE genes were identified by filtering for genes coding for proteins located in the plasma membrane (49). GO terms for biological processes (2021) were computed using enrichR (50). For the analysis of SC cultures, Timepoints 3 to 5 were filtered for genotypes 5, 6, and 10 (Figure 4), while for analysis of SC states these were further filtered by removal of *ITGA5*+ FAP clusters (Figure 5).

#### RNA velocity analysis

RNA velocities were computed for SCs at Timepoint 3 by generating separate count matrices for spliced and unspliced transcripts using velocyto. The resulting loom files were analyzed using scVelo in Python 3.9.12. Transcription kinetics for each individual gene was computed, indicating upregulated expression for a gene when more unspliced transcripts are identified than to be expected from the scVelo dynamical model. A combined velocity graph was generated by reducing the single-gene velocities to a lower-dimensional space using the scv.tl.velocity() function (51, 52).

#### Flow cytometry

For cell type identification, unsorted cells were stained for ITGA5 (PE), ITGA7 (APC), JAM-1 (APC-Cy7), and CD45 (PE-Cy7) (Figure 3; Supplementary Table S3) for 15 min prior to analysis on a MACSQuant10 Flow Analyzer (Miltenyi Biotec). To determine proportions of SCs and FAPs (Figure 4), cells were stained for ITGA7 (APC) and ITGA5 (PE). For analysis of SC states (Figure 5), cells were stained for CD151 (APC) prior to fixation (formaldehyde), permeabilisation (10% saponin), and staining for desmin. Samples were subsequently washed and stained with α-mouse-PE secondary antibodies prior to analysis. Where applicable, unstained and single-stained controls were used for compensation. Cells were pre-gated for size (FSC-A/SSC-A) and singlets (FSC-A/FSC-H), and a minimum of 1×10^4^ events recorded for each sample.

### Fluorescent-activated cell sorting

Cells were purified by FACS 72 h post-isolation on a FACSAria Fusion Cell Sorter (BD Biosciences), following antibody staining as described above. SCs, FAPs, SMMCs, and ECs were sorted using the gating strategy shown in Figure 2. Where noted, cells were gated for NCAM1+/CD29+ (Figure 4G).

### Immunofluorescent staining

After formaldehyde fixation, cells were permeabilised (0.5% Triton X-100), blocked [2% bovine serum albumin (BSA)] and stained with PDGFRα, Pax7, TEK, and CNN1 (for phenotype confirmation; Figure 3C) or desmin (for fusion index; Figure 4C) primary antibodies (see Supplementary Table S3). After washing, cells were incubated with AF488-conjugated secondary antibodies and Hoechst 33342 (Thermo Fisher) prior to imaging on an ImageXpress Pico Automated Cell Imaging System (Molecular Devices).

### Myogenic differentiation assay

Purified cell types (Figure 3) or mixed cultures containing SCs and FAPs in varying proportions (Figure 4) were seeded on 0.5% Matrigel-coated vessels at a density of 5 × 10^4^ cm^−2^ in SFGM for 24 h, before switching to serum-free differentiation media (SFDM) (53). After 72 h in SFDM, cells were fixed with 4% formaldehyde, stained for desmin and imaged as previously described. To calculate fusion indices, number of nuclei within desmin-stained areas were divided by total nuclei.

### Adipogenic differentiation assay

Purified cell types (Figure 3) were seeded on collagen-coated 96-well plates at a density of 5 × 10^4^ cm^−2^ and grown in SFGM for 24 h. To induce adipogenic differentiation, the media was switched to serum-free adipogenic differentiation media (54, 55). Cells were fixed after 240 h and stained for neutral lipids using boron-dipyrromethene (BODIPY, D3922, Thermo Fisher).

### Cell cycle analysis

For flow cytometric cell cycle analysis, SCs were incubated with EdU for 90 min prior to trypsinisation. Following α-CD151-APC staining, Click-iT reaction was performed using the Click-iT EdU Alexa Fluor 488 Flow Cytometry Assay Kit (C10420, Thermo Fisher) according to the supplier’s protocol, followed by α-desmin staining as previously described. Finally, the cells were gated into quiescent (desmin^−^/CD151^+^) and activated (desmin^−^/CD151^−^) and within these, into EdU^+^ (S-Phase), DAPI^low^/EdU^−^ (G0/1-Phase) and DAPI^high^/EdU^−^ (G2-Phase), as shown in Supplementary Figure S6E.

### Statistical analyses

Statistical analyses were performed using Prism 9 (Graphpad). Pearson correlation of fusion indices with percentages of ITGA7+ cells was computed assuming a normal distribution (Figure 4D). For comparisons of culture conditions (Figures 4H–J) and the analysis of cell cycle states in SCs (Figure 5E), two-way ANOVAs were performed including *post-hoc* Tukey’s multiple comparison test. Sample replicates consisted of cells from the same donor animal, cultured in separate vessels. The adjusted *p*-values were denoted as follows: * < 0.05, ** < 0.01, *** < 0.001.

## Data availability statement

The scRNA-seq datasets presented in this study can be found online at: https://www.ncbi.nlm.nih.gov/geo/, accession number GSE211428.

## Ethics statement

Ethical approval was not required for the study involving animals in accordance with the local legislation and institutional requirements because samples were collected from animals being slaughtered to enter the food chain.

## Author contributions

TM, RD, LS, LM, RH, MG, and CD performed experiments and analysis. MP and JF supervised the study. TM and JF wrote the manuscript with input from all authors. All authors contributed to the article and approved the submitted version.

## Conflict of interest

TM, RD, LS, LM, RH, MG, and JF are employees of Mosa Meat B.V. MP is co-founder and stakeholder of Mosa Meat B.V. Study was funded by Mosa Meat B.V. Mosa Meat B.V. has patents on serum-free proliferation medium (WO2021158103), and serum-free myogenic (WO2022114955) and adipogenic differentiation media (WO2023003470).

The remaining author declares that the research was conducted in the absence of any commercial or financial relationships that could be construed as a potential conflict of interest.

## Publisher’s note

All claims expressed in this article are solely those of the authors and do not necessarily represent those of their affiliated organizations, or those of the publisher, the editors and the reviewers. Any product that may be evaluated in this article, or claim that may be made by its manufacturer, is not guaranteed or endorsed by the publisher.

## Supplementary material

SUPPLEMENTARY TABLE S1 Upregulated genes of identified cell types in bovine muscle.
