# Supplementary material for: Single-cell analysis of bovine muscle-derived cell types for cultured meat production
Source: Front Nutr. 2023 Sep 13;10:1212196. doi: 10.3389/fnut.2023.1212196 (PMC10535090; doi:10.3389/fnut.2023.1212196)
Supplement: Supplementary file 2 [file Data_Sheet_1.pdf]

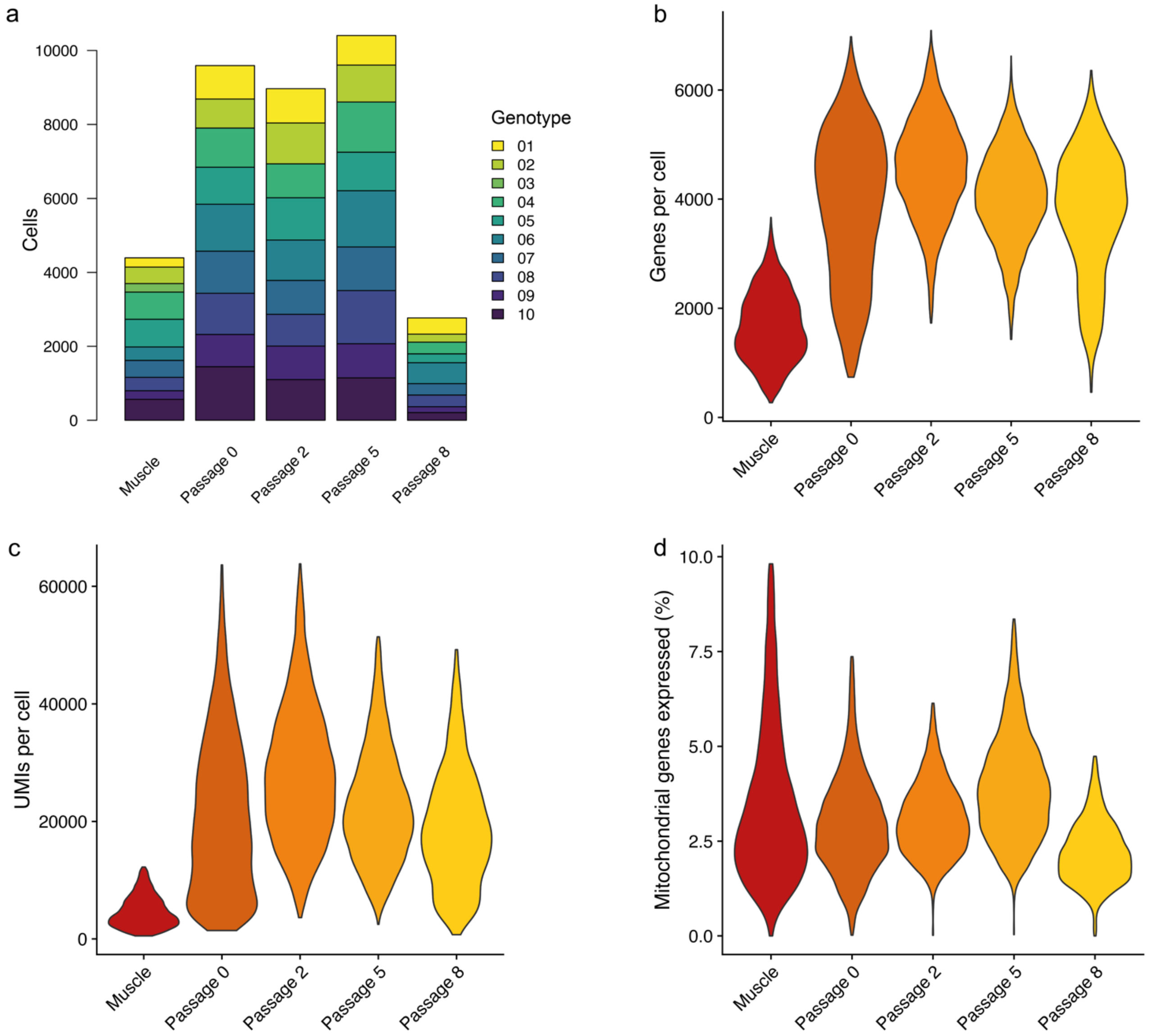

Supplementary Figure 1: Single-cell RNA-sequencing quality control (related to Figure 1)

a) Number of cells at each timepoint of the scRNA-seq experiment, coloured by genotype (donor animal);
b) Total number of genes per cell at each timepoint;
c) Total number of unique molecular identifiers (UMIs) per cell at each timepoint;
d) Percentage of reads assigned to mitochondrial genes at each timepoint.

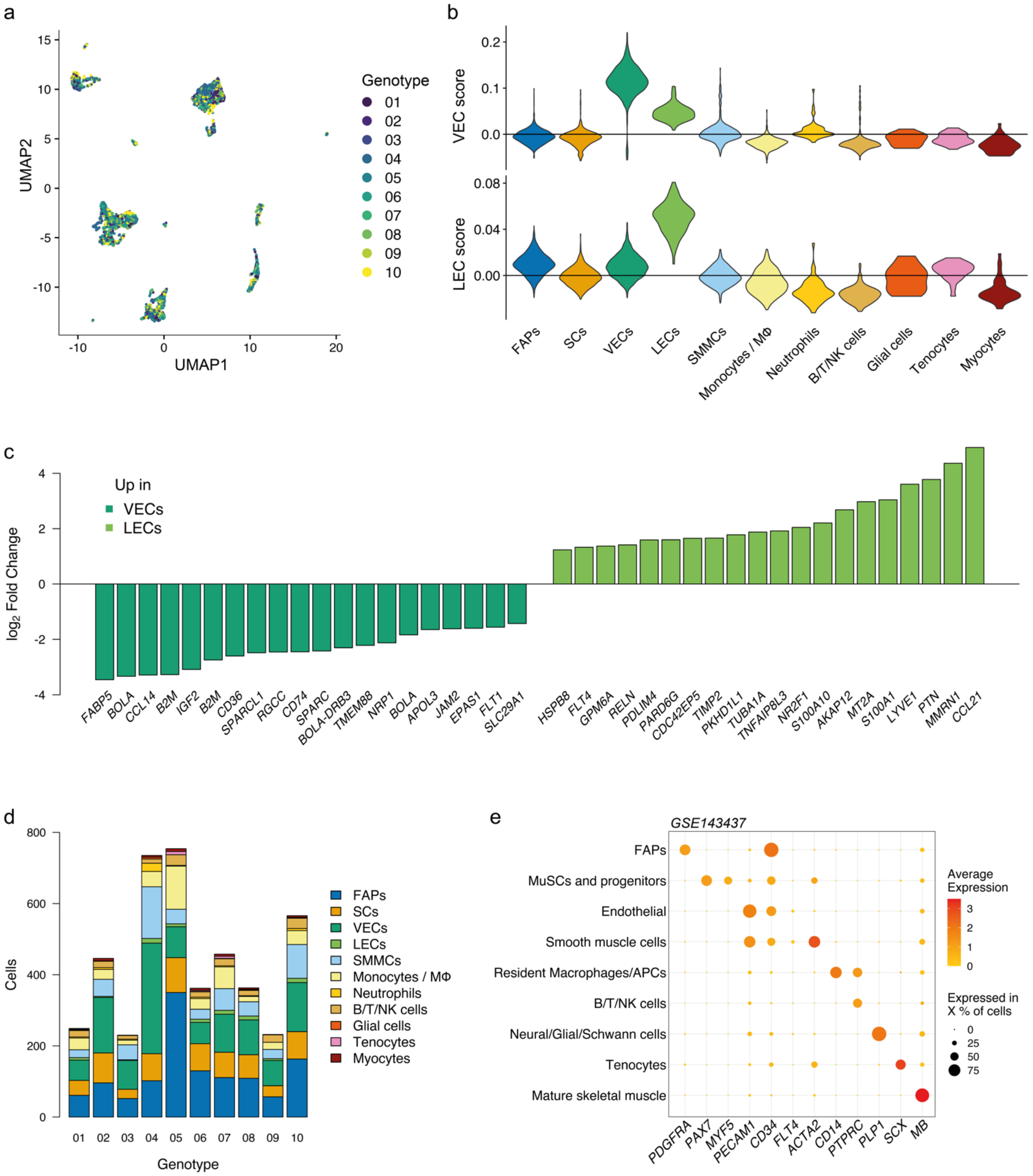

Supplementary Figure 2: Identification of cell types in the bovine muscle niche (related to Figure 1).

a) UMAP of bovine muscle cells coloured by genotype;
b) Averaged expression of *Descartes'* gene signature for vascular (top) and lymphatic endothelial (bottom) marker genes per cluster;
c) Fold changes of the 20 most differentially expressed genes between vascular endothelial cells (VECs, dark green) and lymphatic endothelial cells (LECs, light green);
d) Number of cells per genotype at Timepoint 1, coloured by respective cell type;
e) Dotplot of normalised expression averaged within each cell-type in murine muscle (*GSE143437)* for markers shown in Fig. 1e.

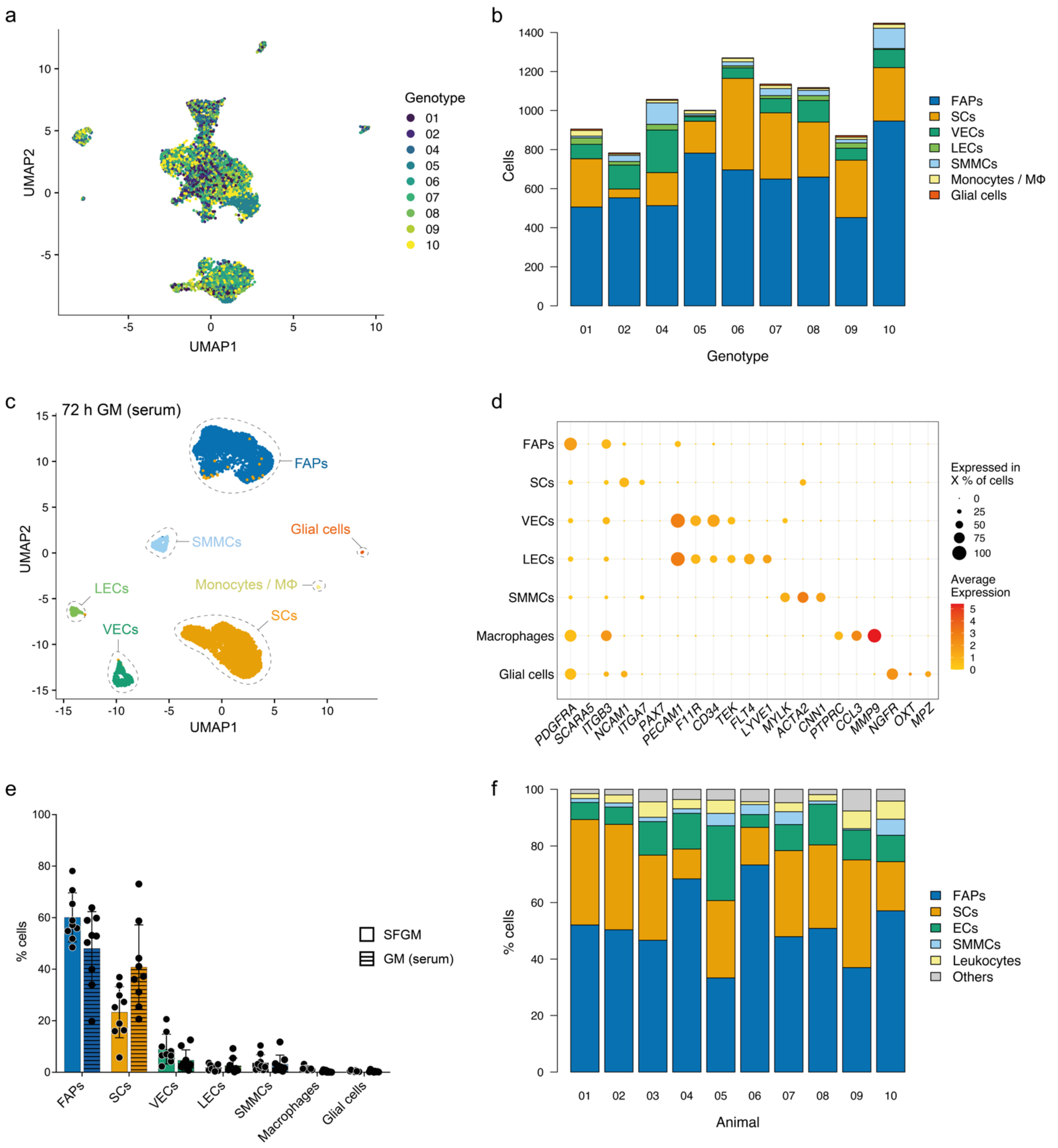

Supplementary Figure 3: Adherent cell types from bovine muscle niche after 72 h in vitro (related to Figure 2).

a) UMAP of adherent cell fraction after 72 h in SFGM, coloured by genotype;
b) Number of cells per genotype at Timepoint 2, coloured by respective cell type;
c) UMAP of adherent cell fraction after 72 h in serum-containing GM, coloured by respective phenotype;
d) Dot plot of marker expression from Fig. 2d in adherent cell fraction after 72 h in serum-containing GM;
e) Comparison of relative cluster sizes of each cell type in serum-containing and serum-free growth media, n = 9;
f) Percentage of cell types in 10 donor animals after 72 h in SFGM, measured via flow cytometry based on gating strategy shown in Fig. 2f.

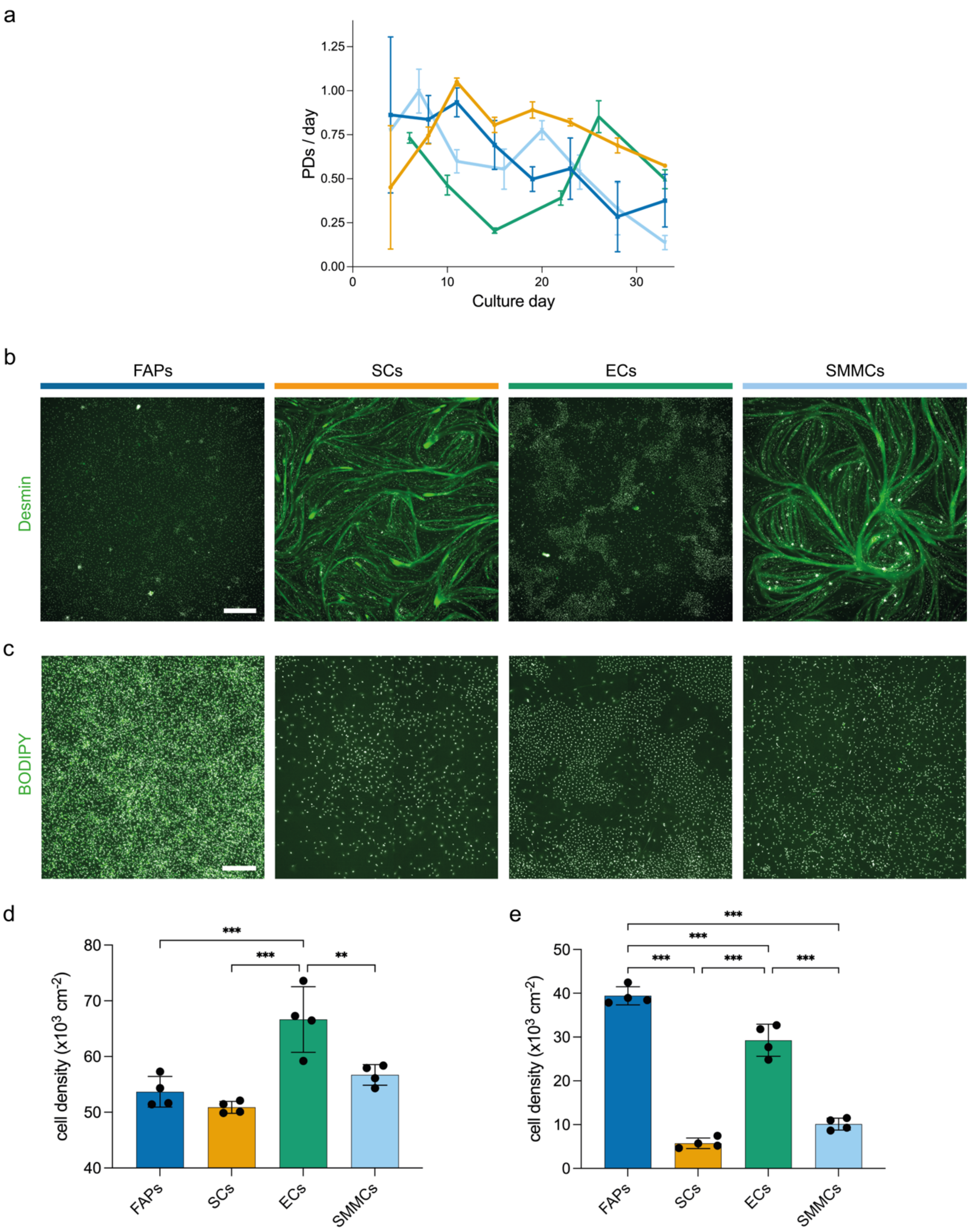

Supplementary Figure 4: Proliferation and differentiation capacities of purified cell types (related to Figure 3).

a) Growth rates during long-term proliferation experiments shown in Fig. 3d. Data is shown as PDs per day; time points correspond to the preceding passage, n = 3;
b) Cell counts for each cell type in myogenic differentiation assay after 72 h in SFDM, n = 4;
c) Cell counts for each cell type after 240 h in serum-free adipogenic differentiation medium, n = 4;
d) Immunofluorescent staining for desmin (green) and Hoechst (white) in purified cell types after 72 h of myogenic differentiation in SFDM; scale bar = 500 µm;
e) Immunofluorescent BODIPY staining (green) in purified cell types after 240 h of adipogenic differentiation; nuclei stained with Hoechst (white); scale bar = 500 µm; Adjusted *p*-values: * < 0.05, ** < 0.01, *** < 0.001.

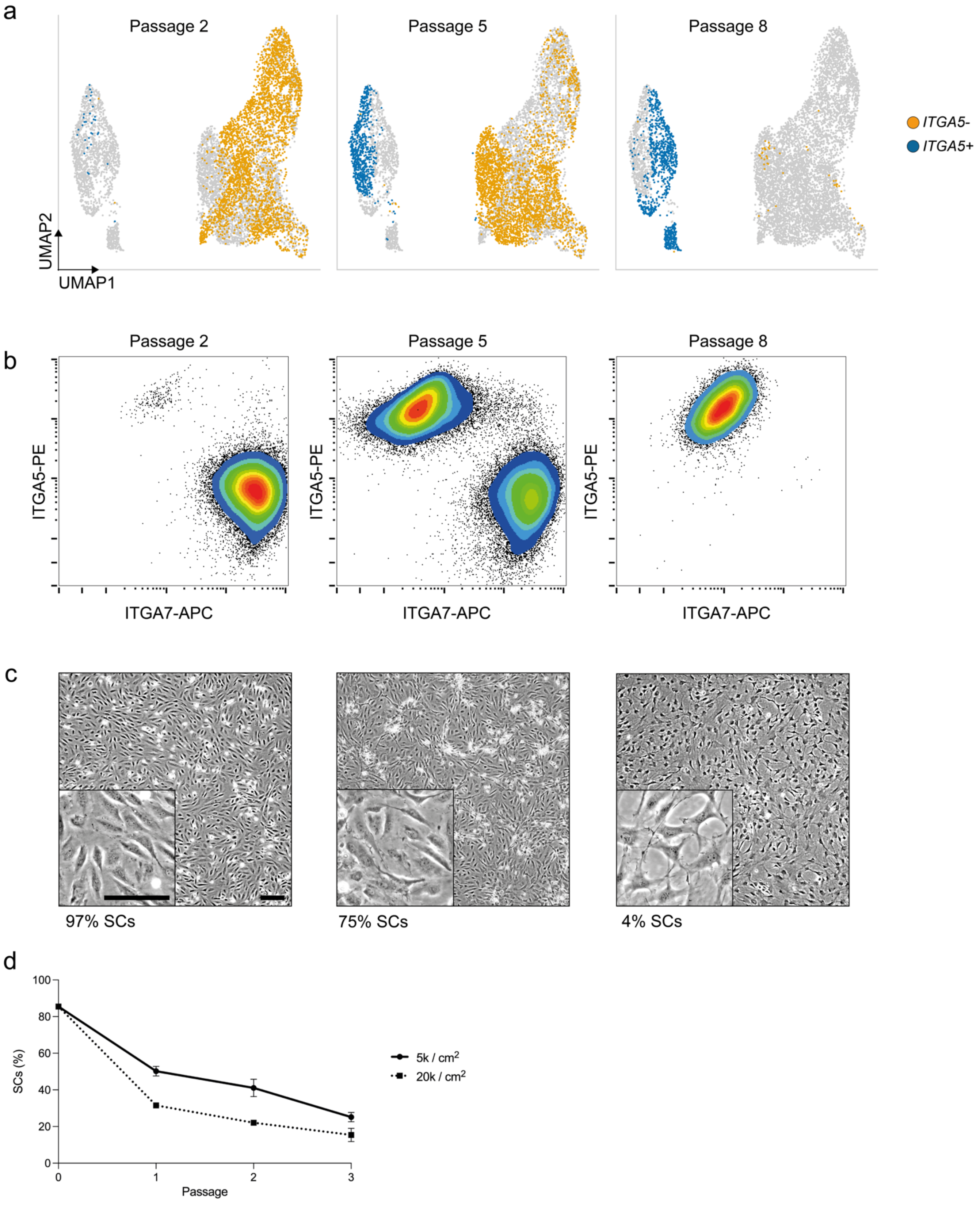

Supplementary Figure 5: Overgrowth of SCs by FAPs during long-term cultivation (related to Figure 4).

a) UMAPs of sorted SCs at passages 2 (Timepoint 3, left), 5 (Timepoint 4, centre) and 8 (Timepoint 5, right), clusters coloured for expression of *ITGA5*;
b) Flow cytometry plots of sorted SCs at passages 2 (left), 5 (centre) and 8 (right);
c) Brightfield images of heterogeneous cultures with denoted ITGA7+ percentages; scale bars = 100 µm;
d) Proportion of SCs resulting from passaging at different seeding densities, as measured via flow cytometry; error bars indicate *SD*, n = 4.

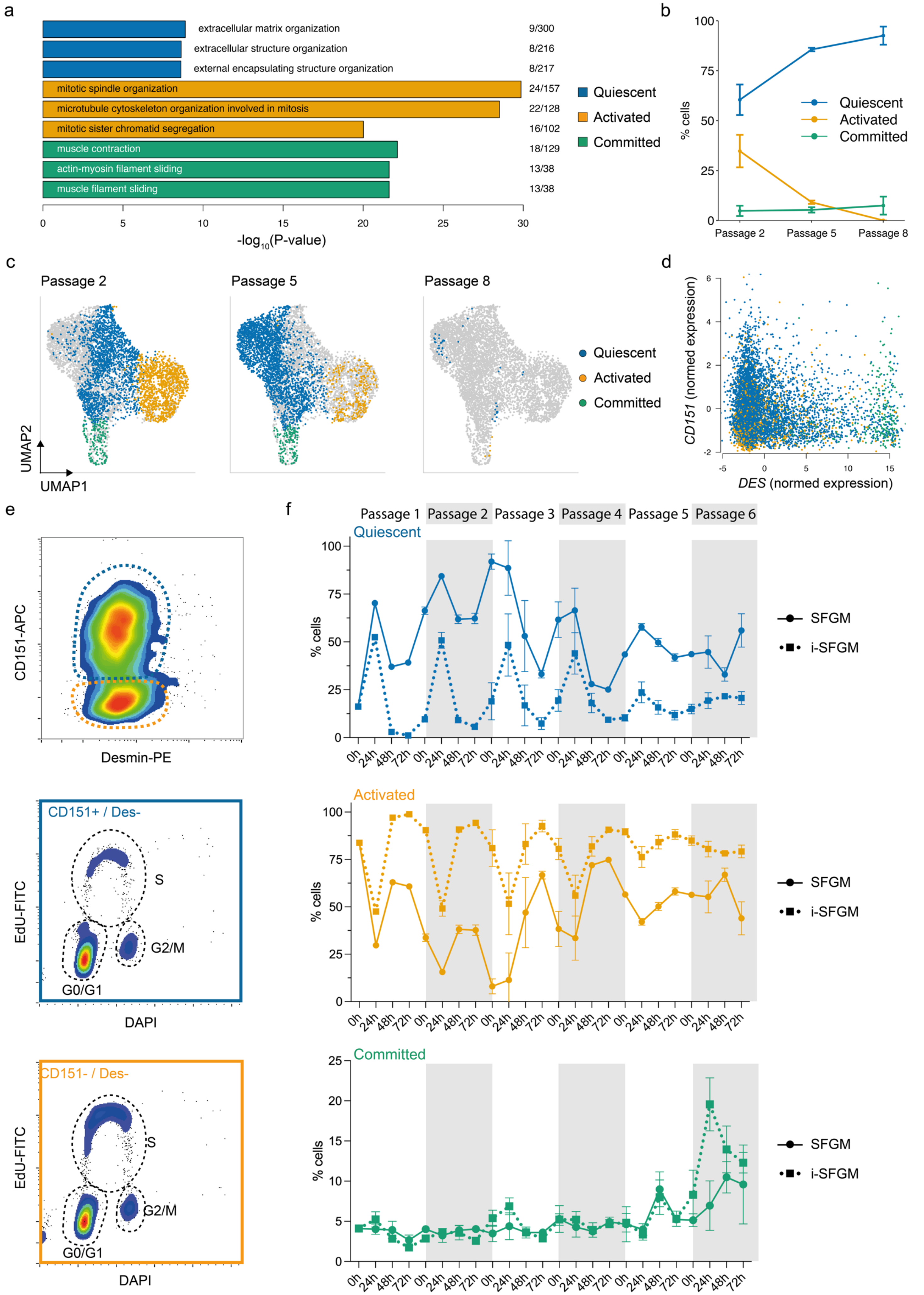

a
extracellular matrix organization 9/300
extracellular structure organization 8/216
external encapsulating structure organization 8/217
mitotic spindle organization 24/157
microtubule cytoskeleton organization involved in mitosis 22/128
mitotic sister chromatid segregation 16/102
muscle contraction 18/129
actin-myosin filament sliding 13/38
muscle filament sliding 13/38
Quiescent
Activated
Committed
-log10(P-value)
b
% cells
Passage 2
Passage 5
Passage 8
Quiescent
Activated
Committed
c
Passage 2
Passage 5
Passage 8
UMAP2
UMAP1
Quiescent
Activated
Committed
d
CD151 (normed expression)
DES (normed expression)
e
CD151-APC
Desmin-PE
CD151+ / Des-
S
G2/M
G0/G1
EdU-FITC
DAPI
CD151- / Des-
S
G2/M
G0/G1
EdU-FITC
DAPI
f
Passage 1
Passage 2
Passage 3
Passage 4
Passage 5
Passage 6
Quiescent
Activated
Committed
% cells
0h 24h 48h 72h
SFGM
i-SFGM

Supplementary Figure 6: Three dynamic states identified within purified SCs (related to Figure 5).

a) Top three most significantly enriched GO terms corresponding to upregulated genes genes in quiescent, activated, and committed SCs;
b) Proportions of quiescent, activated and committed SCs at passages 2, 5 and 8, as determined via scRNA-seq;
c) Combined UMAPs showing SCs at passages 2 (left), 5 (centre) and 8 (right), coloured according to cell state;
d) Expression of *CD151* and *DES* in SCs at passage 2; cells are coloured by state;
e) Gating strategy for cell cycle analysis via flow cytometry; SCs were stained for CD151 and desmin (top); CD151+ (blue, centre) and CD151- (orange, bottom) cells were assigned to cell cycle phases as indicated by dotted gates;
f) Proportion of quiescent (top), activated (centre) and committed (right) SCs in SFGM and improved SFGM media over the course of three passages, determined every 24 h via flow cytometry.

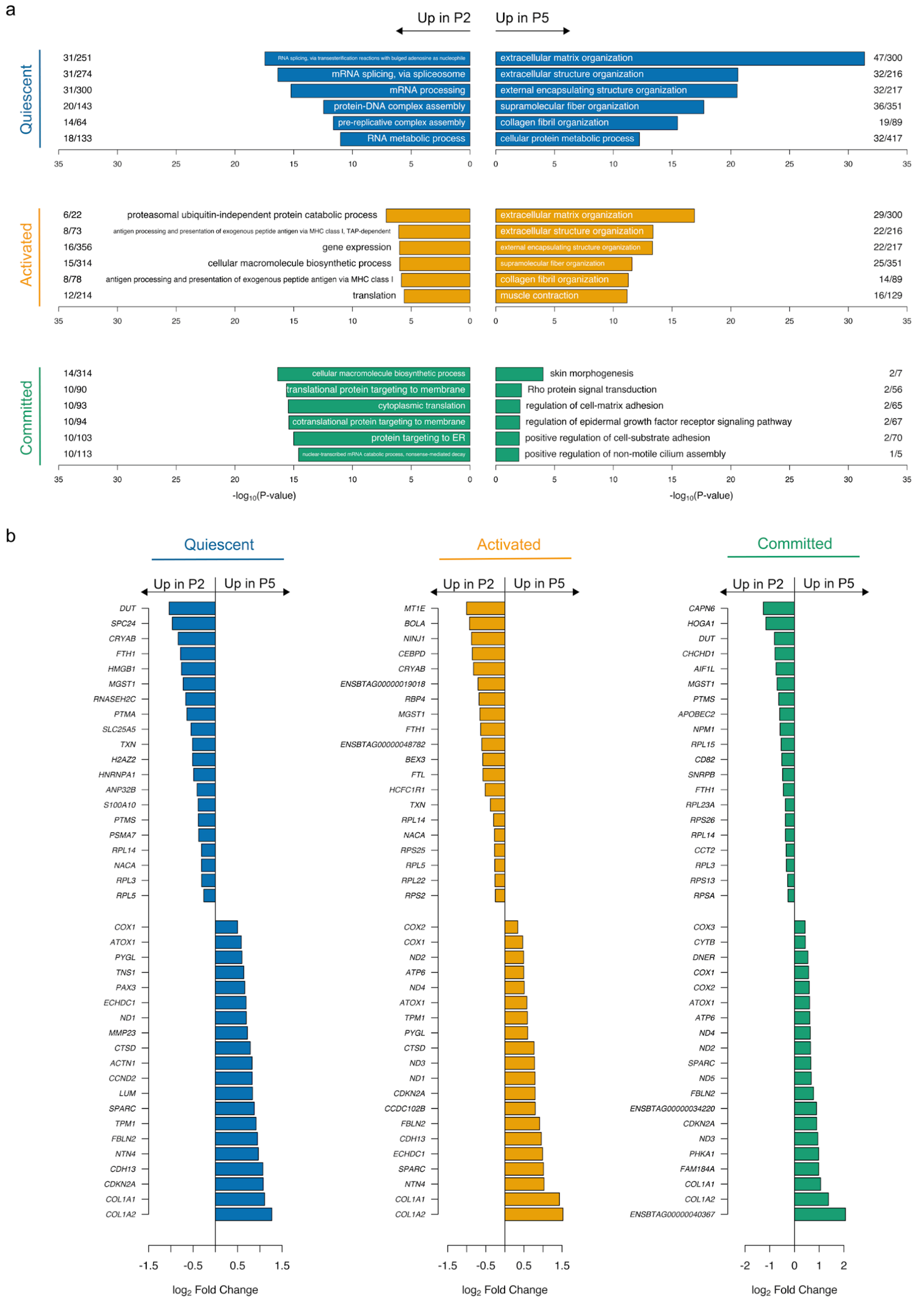

a
Up in P2
Up in P5
Quiescent
31/251 RNA splicing, via transesterification reactions with bulged adenosine as nucleophile
31/274 mRNA splicing, via spliceosome
31/300 mRNA processing
20/143 protein-DNA complex assembly
14/64 pre-replicative complex assembly
18/133 RNA metabolic process
extracellular matrix organization 47/300
extracellular structure organization 32/216
external encapsulating structure organization 32/217
supramolecular fiber organization 36/351
collagen fibril organization 19/89
cellular protein metabolic process 32/417
Activated
6/22 proteasomal ubiquitin-independent protein catabolic process
8/73 antigen processing and presentation of exogenous peptide antigen via MHC class I, TAP-dependent
16/356 gene expression
15/314 cellular macromolecule biosynthetic process
8/78 antigen processing and presentation of exogenous peptide antigen via MHC class I
12/214 translation
extracellular matrix organization 29/300
extracellular structure organization 22/216
external encapsulating structure organization 22/217
supramolecular fiber organization 25/351
collagen fibril organization 14/89
muscle contraction 16/129
Committed
14/314 cellular macromolecule biosynthetic process
10/90 translational protein targeting to membrane
10/93 cytoplasmic translation
10/94 cotranslational protein targeting to membrane
10/103 protein targeting to ER
10/113 nuclear-transcribed mRNA catabolic process, nonsense-mediated decay
skin morphogenesis 2/7
Rho protein signal transduction 2/56
regulation of cell-matrix adhesion 2/65
regulation of epidermal growth factor receptor signaling pathway 2/67
positive regulation of cell-substrate adhesion 2/70
positive regulation of non-motile cilium assembly 1/5
35 30 25 20 15 10 5 0
0 5 10 15 20 25 30 35
-log10(P-value)
b
Quiescent
Activated
Committed
Up in P2
Up in P5
DUT
SPC24
CRYAB
FTH1
HMGB1
MGST1
RNASEH2C
PTMA
SLC25A5
TXN
H2AZ2
HNRNPA1
ANP32B
S100A10
PTMS
PSMA7
RPL14
NACA
RPL3
RPL5
COX1
ATOX1
PYGL
TNS1
PAX3
ECHDC1
ND1
MMP23
CTSD
ACTN1
CCND2
LUM
SPARC
TPM1
FBLN2
NTN4
CDH13
CDKN2A
COL1A1
COL1A2
MT1E
BOLA
NINJ1
CEBPD
CRYAB
ENSBTAG00000019018
RBP4
MGST1
FTH1
ENSBTAG00000048782
BEX3
FTL
HCFC1R1
TXN
RPL14
NACA
RPS25
RPL5
RPL22
RPS2
COX2
COX1
ND2
ATP6
ND4
ATOX1
TPM1
PYGL
CTSD
ND3
ND1
CDKN2A
CCDC102B
FBLN2
CDH13
ECHDC1
SPARC
NTN4
COL1A1
COL1A2
CAPN6
HOGA1
DUT
CHCHD1
AIF1L
MGST1
PTMS
APOBEC2
NPM1
RPL15
CD82
SNRPB
FTH1
RPL23A
RPS26
RPL14
CCT2
RPL3
RPS13
RPSA
COX3
CYTB
DNER
COX1
COX2
ATOX1
ATP6
ND4
ND2
SPARC
ND5
FBLN2
ENSBTAG00000034220
CDKN2A
ND3
PHKA1
FAM184A
COL1A1
COL1A2
ENSBTAG00000040367
-1.5 -0.5 0.5 1.5
-2 -1 0 1 2
log2 Fold Change

Supplementary Figure 7: Aging effects within SC subpopulations (related to Figure 5).

a) Most significantly enriched GO-terms corresponding to the 200 most upregulated genes between passage 2 (left) and passage 5 (right) in quiescent (blue), activated (orange), and committed (green) SCs;
b) Fold changes of the 20 most up- and downregulated genes in quiescent (blue), activated (orange), and committed (green) SCs.

Supplementary Table 2: Media formulations.

#	Component	Reference	Concentration
**Serum-free growth medium (SFGM)**			
1	DMEM/F-12	21331-020, Gibco	
2	α-linolenic acid	L2376, Sigma Aldrich	1.0 μg ml^{-1}
3	FGF-2	100-18B, Peprotech	10 ng ml^{-1}
4	Human Serum Albumin	Rc HA NW20, Richcore Lifesciences	5.0 mg ml^{-1}
5	HGF	100-39H, Peprotech	5 ng ml^{-1}
6	Hydrocortisone	H0888, Sigma Aldrich	36 ng ml^{-1}
7	IGF-1	100-11, Peprotech	100 ng ml^{-1}
8	IL-6	200-06, Peprotech	20 ng ml^{-1}
9	ITSE	00-101, biogems	1%
10	GlutaMax	35050-061, Gibco	1%
11	Glucose	G7021, Sigma Aldrich	17.7 mM
12	L-ascorbic acid 2-phosphate (Vitamin C)	A8960, Sigma Aldrich	155 μM
13	PDGF-BB	100-14B, Peprotech	10 ng ml^{-1}
14	Penicillin/Streptomycin/Amphotericin (PSA)	17-745E, Lonza	1%
15	VEGF	100-20 Peprotech	10 ng ml^{-1}
**Growth medium (GM)**			
1	Ham’s F-10 Nutrient Mix	31550-023, Gibco	
2	Fetal Bovine Serum, heat inactivated (FBS)	10082147, Gibco	20%
3	FGF-2	100-18B, Peprotech	5 ng ml^{-1}
4	PSA	17-745E, Lonza	1%
**Serum-free myogenic differentiation medium (SFDM)**			
1	DMEM/F-12	21311-020, Gibco	
2	EGF-1	AF-100-15, Peprotech	10 ng ml^{-1}
3	Human Serum Albumin	Rc HA NW20, Richcore Lifesciences	0.5 mg ml^{-1}
4	ITSE	00-101, biogems	2%
5	L-ascorbic acid 2-phosphate (Vitamin C)	A8960, Sigma Aldrich	40 μM
6	Lysophosphatidic acid (LPA)	L7260, Sigma Aldrich	1 μM
7	MEM Amino Acids Solution	11130-051, ThermoFisher	0.50%
8	$NaHCO_3$	P2256, Sigma Aldrich	6.5 mM
9	PSA	17-745E, Lonza	1%
10	Soy hydrolysates	58903C, Merck	1%
**Serum-free adipogenic differentiation medium**			
1	DMEM/F-12	21311-020, Gibco	
2	CaCl2	C3881, Sigma Aldrich	1 mM
3	D-(+)-Galactose	G5388, Sigma Aldrich	17 mM
4	Hydrocortisone	H0888, Sigma Aldrich	9 ng ml^{-1}
5	Indomethacin	I7378, Sigma Aldrich	5 nM

6	Insulin (recombinant human)	I0516, Sigma Aldrich	10 µg ml^{-1}
7	L-ascorbic acid 2-phosphate (Vitamin C)	A8960, Sigma Aldrich	227 µM
8	Chemically defined lipid concentrate	11905-031, Gibco	0.1%
9	PSA	17-745E, Lonza	1%
10	Putrescine dihydrochloride	51799, Sigma Aldrich	57 µM
11	Sodium pyruvate	P2256, Sigma Aldrich	10 mM
12	Water (Type 1)	Millipore, Merck	15%

Supplementary Table 3: Antibodies used in this study.

Target	Colour	Source	Dilution	Reference	Application
Calponin-1	-	Abcam	1:400	ab46794	IF
CD151	APC	Miltenyi Biotec	1:50	130-103-664	Flow
CD29	APC	BioLegend	1:20	B247653	Flow
CD45-Ro	APC-Vio770	Miltenyi Biotec	1:50	130-114-083	Flow
Desmin	-	Sigma Aldrich	1:1000	D1033	Flow; IF
ITGA5	PE	Miltenyi Biotec	1:50	130-110-532	Flow
ITGA7	APC	Miltenyi Biotec	1:50	130-123-833	Flow
JAM-1 / F11R	PE-Vio770	Miltenyi Biotec	1:50	130-109-484	Flow
NCAM1	PE	BD Biosciences	1:20	335826	Flow
Pax7	-	Developmental Studies Hybridoma Bank	1:100	Cat# PAX7	IF
PDGFRα	-	Abcam	1:200	ab203491	IF
TEK / Tie2	-	BioLegend	1:100	334202	IF
mouse	AF488	Invitrogen	1:250	A-11029	IF
mouse	PE	Miltenyi Biotec	1:250	30-119-684	IF
rabbit	AF488	Invitrogen	1:250	A-11034	IF
